# Usefulness of the mini nutritional assessment short-form for evaluating nutritional status in patients with nontuberculous mycobacterial pulmonary disease: a prospective cross-sectional study

**DOI:** 10.1186/s12879-024-09499-3

**Published:** 2024-06-19

**Authors:** Eunki Chung, Youngmok Park, Hye-Jeong Lee, Young Ae Kang

**Affiliations:** 1 Division of Pulmonology, Department of Internal Medicine, National Health Insurance Service Ilsan Hospital, Goyang, Republic of Korea; 2 Yonsei University Graduate School of Medicine, Seoul, Republic of Korea; 3 Division of Pulmonary and Critical Care Medicine, Department of Internal Medicine, Severance Hospital, Yonsei University College of Medicine, 50-1 Yonsei-ro, Seodaemun-gu, Seoul, 03722 Republic of Korea; 4 Department of Radiology, Research Institute of Radiological Science, Severance Hospital, Yonsei University College of Medicine, Seoul, Republic of Korea; 5 Institute for Immunology and Immunological Disease, Yonsei University College of Medicine, Seoul, Republic of Korea

**Keywords:** Mini Nutritional Assessment Short-Form, Nontuberculous mycobacteria, Nutritional status

## Abstract

**Background:**

Although the Mini Nutritional Assessment (MNA) is recognized as a useful tool for evaluating nutritional status in patients with various diseases, its applicability in patients with nontuberculous mycobacterial pulmonary disease (NTM-PD) remains undetermined.

**Methods:**

We designed a prospective cross-sectional study to investigate whether the MNA Short-Form (MNA-SF) score can serve as a screening tool to assess the nutritional status of patients with NTM-PD. The MNA-SF was conducted upon patient enrollment, and correlation analyses were performed to compare MNA-SF scores with other nutritional measurements and disease severity. Multivariable logistic regression analyses were conducted to evaluate the association between MNA-SF scores and NTM-PD severity.

**Results:**

The 194 patients with NTM-PD included in the analysis had a median age of 65.0 (59.0–69.0) years; 59.3% (*n* = 115) had low MNA-SF scores (< 12). The low MNA-SF group exhibited a lower body mass index (19.7 vs. 22.4 kg/m^2^, *p* < 0.001) and fat-free mass index (14.7 vs. 15.6 kg/m^2^, *p* < 0.001) than the normal MNA-SF group, as well as higher incidences of sarcopenia (20.0% vs. 6.3%, *p* = 0.008) and adipopenia (35.7% vs. 5.1%, *p* < 0.001). However, no significant differences in calorie and protein intakes were observed between the two groups. Low MNA-SF scores were associated with radiographic severity (adjusted odds ratio 2.72, 95% confidence interval 1.38–5.36) but not with forced vital capacity.

**Conclusions:**

The MNA-SF can effectively assess the nutritional status of patients with NTM-PD and can serve as an important clinical indicator in NTM-PD where treatment timing is determined by clinical judgment.

**Supplementary Information:**

The online version contains supplementary material available at 10.1186/s12879-024-09499-3.

## Background

Nontuberculous mycobacterial pulmonary disease (NTM-PD) is a chronic respiratory infection, the incidence of which is increasing globally [1]. Therapeutic failures and relapses are common, causing progressive lung damage that eventually results in a decline in lung function and quality of life [2–4]. In elderly slender women, NTM-PD is known to frequently occur [5], and low body mass index (BMI) has been identified as a risk factor of NTM-PD incidence and mortality [6, 7]. Consequently, malnutrition may be prevalent in patients with NTM-PD. Although one preliminary study has investigated the nutritional status of female patients with NTM-PD [8], nutritional assessments are not routinely performed in clinical settings.

The nutritional status of patients with respiratory diseases can be evaluated using various methods, including BMI [9, 10]; fat-free mass index (FFMI), which is the fat-free mass in kg divided by the height in meters squared, with body fat estimated by measuring triceps skinfold thickness; and the prognostic nutritional index, which is calculated through laboratory analysis of blood samples [11–13]. Some of these methods can be challenging to implement routinely in clinical settings because of their complexity and associated costs. The Mini Nutritional Assessment (MNA) is a simple and effective method for assessing the nutritional status of older patients [14] that has been validated in other respiratory diseases and may therefore also be applicable to patients with NTM-PD [15–17]. As patients with NTM-PD are often older, use of the MNA Short-Form (MNA-SF), which involves fewer questions, may be more suitable. The MNA-SF evaluates dietary intake, recent changes in weight, neuropsychological issues, and functional abilities through a brief survey, combining this information with BMI. If BMI measurement is not possible, calf circumference can be used as a substitute [18]. Few studies have analyzed nutritional screening methods in patients with NTM-PD. Therefore, we investigated whether MNA-SF can be utilized for assessing the nutritional status of patients with NTM-PD. Additionally, we examined the association between nutritional status, as assessed using the MNA-SF, and disease severity.

## Methods

### Study design and population

This prospective cross-sectional study recruited patients with NTM-PD between January and October 2022 at a tertiary referral hospital in the Republic of Korea. Patients aged ≥ 19 years diagnosed with NTM-PD according to the guidelines issued by the American Thoracic Society and the Infectious Diseases Society of America [19] and who agreed to participate in the study were initially enrolled, irrespective of their treatment status. All 194 registered individuals were included in the analysis.

### Data collection and measurement

#### MNA-SF

The MNA-SF was performed upon enrollment. A score was assigned for each of the six questions on dietary intake, weight loss, mobility, psychological stress or acute disease, neuropsychological problems, and BMI or calf circumference. The total possible score was 14 points; a score of 12 or higher indicated normal nutritional status, and a score of less than 12 indicated that patients were at risk of malnutrition (8–11 points) or malnourished (0–7 points). The content and scoring system of the MNA-SF questionnaire are provided in Supplementary Table S1 [18].

#### Nutritional intake assessment

Dietary intake was examined upon enrollment using the 24-h recall method, which involved respondents recalling and reporting all food and beverages consumed over a 24-h period [20]. The total calorie intake was compared with the calorie requirement as calculated by the Estimated Energy Requirement (EER), taking into account activity factor; the Reference Nutrient Intake (RNI) for older adults was used to determine the amount of protein required [21, 22].

### Disease severity

The severity of NTM-PD was assessed using computed tomography (CT). CT images were analyzed for bronchiectasis, cellular bronchiolitis, nodules, cavities, and consolidation, according to a previously published scoring system [23]. Non-contrast chest CT was performed within 1 year of enrollment. Out of a total possible score of 30 points, a score of ≥ 10 points was considered to indicate severe disease. CT evaluation was conducted by two experienced radiologists with more than 10 years’ expertise, who determined the scores without reference to clinical information.

### Respiratory symptoms and quality of life

NTM-PD symptoms (cough, sputum, and tightness) and patient quality of life were measured using the chronic obstructive pulmonary disease (COPD) assessment test (CAT), which comprises eight domains and is scored using a 5-point Likert scale [24]. The total CAT score was obtained by summing the scores from all eight domains; a CAT score ≥ 10 was defined as a High CAT score. The domain-specific scores for the three symptoms were also assessed separately.

#### Sarcopenia and Adipopenia

Possible sarcopenia and sarcopenia were defined according to the criteria proposed by the Asian Working Group for Sarcopenia. Possible sarcopenia was defined as low handgrip strength (< 28 kg for males and < 18 kg for females); sarcopenia was diagnosed when patients also exhibited low appendicular skeletal muscle mass divided by height squared (< 7.0 kg/m^2^ for males and < 5.7 kg/m^2^ for females) [25]. Adipopenia was defined when the fat mass index (FMI) was below the lower limit of the normal range (< 3 kg/m^2^ for males and < 5 kg/m^2^ for females), based on a study examining normal FMI in a Korean population [26]. Bioelectrical impedance analysis (BIA) was performed using an InBody 770 device (Biospace Co. Ltd., Seoul, Republic of Korea) to evaluate the body composition of patients with NTM-PD.

### Statistical analysis

Statistical analyses were performed using R software version 4.2.1 (R Foundation for Statistical Computing, Vienna, Austria) and SPSS software version 26.0 (IBM Corp., Armonk, NY, USA). Descriptive variables are summarized using medians and interquartile ranges (IQR), means and standard deviations, or frequencies and percentages. Chi-squared and Fisher’s exact tests were used to compare categorical variables, and Student’s t-tests and Mann–Whitney U tests were used to compare continuous variables. Since there were no prior studies evaluating nutritional status using the MNA-SF for NTM-PD, we used the malnutrition rate of 65% from a previous study that assessed nutritional status using the MNA-SF in patients with COPD [27]. When we assumed the 95% confidence interval and the width of a 20% confidence interval, we needed 87 participants to assess the nutritional status. Considering an anticipated dropout rate of 10%, the final sample size was calculated to be 109 participants. Correlation analyses were conducted using Spearman’s correlation for CT and MNA-SF scores and Pearson’s correlation coefficient for MNA-SF scores and other variables. Univariable and multivariable logistic regression analyses were used to investigate the association between the MNA-SF score and NTM-PD severity. Restrictive cubic spline curves were used to analyze the association between the MNA-SF score and the adjusted odds ratio (aOR) of the CT score. A *P*-value < 0.05 was considered statistically significant.

## Results

### Baseline characteristics of participants

Table 1 presents the baseline characteristics of participants according to sex. The median age of the participants was 65.0 (IQR 59.0–69.0) years, and the proportion of females (71.6%) was higher than that of males. A history of tuberculosis was the most common comorbidity (22.2%), followed by cancer (14.9%). Regarding radiographic severity, the prevalence of cavities was 35.1% (38.2% in males and 33.8% in females), and 32.0% of participants had a CT score ≥ 10. For acid-fast bacilli (AFB) positivity reflecting microbiological severity, accounted for 16.5%, and the median values of the MNA-SF score, CAT score, and BMI were 11.0 (IQR 9.0–12.0), 11.0 (IQR 5.0–18.0), and 20.9 (IQR 19.4–22.8) kg/m^2^, respectively. The mean total calorie intake represented 96.0% of the EER; however, the median protein intake represented 85.0% of the RNI, indicating relatively insufficient protein intake by patients with NTM-PD.

**Table 1:** Baseline patient characteristics by sex

Characteristics	Total participant (*N* = 194)	Male (*N* = 55)	Female (*N* = 139)	*p*-value
**Age (year)**	65.0 (59.0–69.0)	68.0 (62.0–71.0)	63.0 (58.0–67.0)	0.001
**Smoking status (Current or former), no. (%)**	34 (17.5)	28 (50.9)	6 (4.3)	< 0.001
**BMI (kg/m** ^**2**^ **)**	20.9 (19.4–22.8)	20.9 (19.2–23.7)	20.9 (19.5–22.4)	0.794
**FFMI (kg/m** ^**2**^ **)**	14.9 (14.2–16.2)	16.5 (15.4–18.1)	14.6 (13.9–15.3)	< 0.001
**Comorbidities**				
History of TB, no. (%)	43 (22.2)	10 (18.2)	33 (23.7)	0.401
COPD, no. (%)	14 (7.2)	6 (10.9)	8 (5.8)	0.211
Asthma, no. (%)	9 (4.6)	3 (5.5)	6 (4.3)	0.715
DM, no. (%)	22 (11.3)	9 (16.4)	13 (9.4)	0.165
CV, no. (%)	19 (9.8)	6 (10.9)	13 (9.4)	0.742
Cancer, no. (%)	29 (14.9)	14 (25.5)	15 (10.8)	0.010
**Treatment ≥ 1, no. (%)**	102 (52.6)	30 (54.5)	72 (51.8)	0.730
**Cavity ≥ 1, no. (%)**	68 (35.1)	21 (38.2)	47 (33.8)	0.565
**CT score**	7.5 (6.0–10.3)	7.0 (5.0–10.0)	8.0 (6.0–11.0)	0.316
**CT score ≥ 10, no. (%)**	62 (32.0)	17 (30.9)	45 (32.4)	0.844
**AFB positivity, no. (%)**	32 (16.5)	12 (21.8)	20 (14.4)	0.209
**Handgrip strength (kg)**	22.2 (19.4–26.6)	30.0 (25.5–33.9)	20.9 (18.2–23.6)	< 0.001
Cough*	1.0 (0.0–3.0)	1.0 (1.0–2.0)	1.0 (0.0–3.0)	0.485
Sputum*	2.0 (1.0–3.0)	2.0 (1.0–3.0)	2.0 (1.0–3.0)	0.680
Tightness*	1.0 (0.0–2.0)	1.0 (0.0–2.0)	1.0 (0.0–2.0)	0.497
**CAT score**	11.0 (5.0–18.0)	12.0 (6.0–20.0)	11.0 (4.0–17.0)	0.343
**MNA-SF score**	11.0 (9.0–12.0)	11.0 (9.0–13.0)	11.0 (10.0–12.0)	0.990
**Possible sarcopenia, no. (%)**	48 (24.7)	20 (36.4)	28 (20.1)	0.018
**Sarcopenia, no. (%)**	28 (14.4)	16 (29.1)	12 (8.6)	< 0.001
**Adipopenia, no. (%)**	45 (23.2)	11 (20.0)	34 (24.5)	0.507
**Body composition**				
Skeletal muscle mass (kg)	20.5 (18.2–23.2)	26.7 (23.3–29.1)	19.3 (17.7–21.1)	< 0.001
ASM/m^2^ (kg/m^2^)	6.0 (5.6–6.7)	7.2 (6.6–7.9)	5.8 (5.5–6.3)	< 0.001
Body fat mass (kg)	15.1 ± 5.9	12.7 ± 5.2	16.1 ± 5.9	< 0.001
FMI (kg/m^2^)	6.0 ± 2.5	4.5 ± 1.9	6.5 ± 2.4	< 0.001
Fat percent (%)	27.1 ± 8.1	20.4 ± 6.6	29.7 ± 7.0	< 0.001
**Total calorie intake (kcal/day)**	1374.3 ± 387.3	1493.8 ± 405.4	1327.1 ± 370.8	0.007
**Total calorie intake (%EER)**	96.0 ± 27.8	88.1 ± 25.0	99.2 ± 28.3	0.012
**Protein intake (g/day)**	54.9 (42.8–70.7)	62.0 (45.5–77.7)	52.6 (40.8–65.5)	0.011
**Protein intake (%RNI)**	85.0 (65.6–109.8)	87.9 (62.3–104.0)	83.2 (65.8–113.5)	0.470
FVC (%)**	85.9 (76.6–98.9)	96.2 (78.3–119.4)	84.2 (75.6–91.9)	0.003
FVC < 80%, no. (%) **	45 (37.2)	10 (27.8)	35 (41.2)	0.163

Data are presented as number (%), median (interquartile range), or means ± standard deviation

* 187 patients included (55 males and 132 females)

** 121 patients included (36 males and 85 females)

BMI, body mass index; FFMI, fat-free mass index; TB, tuberculosis; COPD, chronic obstructive pulmonary disease; DM, diabetes mellitus; CV, cardiovascular disease; CT, computed tomography; AFB, acid-fast bacilli; CAT, chronic obstructive pulmonary disease assessment test; MNA-SF, Mini Nutritional Assessment Short Form; ASM, appendicular skeletal muscle mass; FMI, fat mass index; %EER, percentage of estimated energy requirement; %RNI, percentage of recommended nutrient intake; FVC, forced vital capacity

Table 2 presents the differences in characteristics between patients with a normal MNA-SF score (≥ 12) and those with a low MNA-SF score (< 12). The low MNA-SF group included 115 (59.3%) patients, of whom 14 (7.2%) were malnourished and 101 (52.1%) were at risk of malnutrition. No statistically significant differences were observed between the normal and low MNA-SF groups in terms of age, sex, and comorbidities. However, the median CT score and the proportion of patients with a CT score ≥ 10 were higher in the low MNA-SF group than in the normal MNA-SF group (8.0 vs. 6.0, *p* = 0.003 and 40.0 vs. 20.3%, *p* = 0.004, respectively), suggesting that patients with low MNA-SF scores had more severe disease. The low MNA-SF group had a lower BMI (19.7 vs. 22.4 kg/m^2^, *p* < 0.001) and FFMI (14.7 vs. 15.6 kg/m^2^, *p* < 0.001) than the normal MNA-SF group, but no significant differences were observed in total calorie or protein intakes. All body composition measurements were lower in the low MNA-SF group than in the normal MNA-SF group. The prevalence of sarcopenia and adipopenia were higher in the low MNA-SF group than in the normal MNA-SF group (20.0% vs. 6.3%, *p* = 0.008 and 35.7% vs. 5.1%, *p* < 0.001, respectively).

**Table 2:** Baseline patient characteristics by MNA-SF score (normal, ≥ 12; low, < 12)

Characteristics	Total participant (*N* = 194)	Normal MNA-SF (*N* = 79)	Low MNA-SF (*N* = 115)	*p*-value
**Age (year)**	65.0 (59.0–69.0)	66.0 (60.0–69.0)	63.0 (58.0–69.0)	0.182
**Female, no. (%)**	139 (71.6)	58 (73.4)	81 (70.4)	0.651
**Smoking status (Current or former), no. (%)**	34 (17.5)	17 (21.5)	17 (14.8)	0.225
**BMI (kg/m** ^**2**^ **)**	20.9 (19.4–22.8)	22.4 (20.8–24.1)	19.7 (18.6–21.2)	< 0.001
**FFMI (kg/m** ^**2**^ **)**	14.9 (14.2–16.2)	15.6 (14.5–17.0)	14.7 (13.9–15.7)	< 0.001
**Comorbidities**				
History of TB, no. (%)	43 (22.2)	13 (16.5)	30 (26.1)	0.113
COPD, no. (%)	14 (7.2)	9 (11.4)	5 (4.3)	0.062
Asthma, no. (%)	9 (4.6)	4 (5.1)	5 (4.3)	1.000
DM, no. (%)	22 (11.3)	9 (11.4)	13 (11.3)	0.985
CV, no. (%)	19 (9.8)	5 (6.3)	14 (12.2)	0.178
Cancer, no. (%)	29 (14.9)	14 (17.7)	15 (13.0)	0.369
**Treatment ≥ 1, no. (%)**	102 (52.6)	36 (45.6)	66 (57.4)	0.105
**Cavity ≥ 1, no. (%)**	68 (35.1)	27 (34.2)	41 (35.7)	0.832
**CT score**	7.5 (6.0–10.3)	6.0 (5.0–9.0)	8.0 (6.0–12.0)	0.003
**CT score ≥ 10, no. (%)**	62 (32.0)	16 (20.3)	46 (40.0)	0.004
**AFB positivity, no. (%)**	32 (16.5)	12 (15.2)	20 (17.4)	0.685
**Handgrip strength (kg)**	22.2 (19.4–26.6)	22.0 (19.4–27.2)	22.3 (19.4–26.2)	0.758
Cough*	1.0 (0.0–3.0)	1.0 (0.0–2.0)	1.0 (0.0–3.0)	0.999
Sputum*	2.0 (1.0–3.0)	1.0 (1.0–3.0)	2.0 (1.0–3.0)	0.661
Tightness*	1.0 (0.0–2.0)	1.0 (0.0–2.0)	1.0 (0.0–2.0)	0.494
**CAT score**	11.0 (5.0–18.0)	10.0 (4.0–18.0)	12.0 (7.0–18.0)	0.576
**Possible sarcopenia, no. (%)**	48 (24.7)	13 (16.5)	35 (30.4)	0.027
**Sarcopenia, no. (%)**	28 (14.4)	5 (6.3)	23 (20.0)	0.008
**Adipopenia, no. (%)**	45 (23.2)	4 (5.1)	41 (35.7)	< 0.001
**Body composition**				
Skeletal muscle mass (kg)	20.5 (18.2–23.2)	21.1 (18.6–26.0)	20.2 (17.9–22.4)	0.039
ASM/m^2^ (kg/m^2^)	6.0 (5.6–6.7)	6.4 (5.7–7.3)	6.0 (5.5–6.5)	0.004
Body fat mass (kg)	15.1 ± 5.9	18.4 ± 5.9	12.9 ± 4.8	< 0.001
FMI (kg/m^2^)	6.0 ± 2.5	7.2 ± 2.6	5.1 ± 1.9	< 0.001
Fat percent (%)	27.1 ± 8.1	30.6 ± 7.6	24.6 ± 7.5	< 0.001
**Total calorie intake (kcal/day)**	1374.3 ± 387.3	1419.2 ± 386.0	1343.5 ± 386.8	0.182
**Total calorie intake (%EER)**	96.0 ± 27.8	96.5 ± 28.6	95.7 ± 27.3	0.843
**Protein intake (g/day)**	54.9 (42.8–70.7)	55.5 (43.0-74.2)	53.4 (42.7–68.5)	0.514
**Protein intake (%RNI)**	85.0 (65.6–109.8)	88.8 (65.2-114.9)	81.4 (65.8-109.2)	0.467
FVC (%) **	87.1 ± 19.9	86.7 ± 18.9	87.4 ± 20.7	0.837
FVC < 80%, no. (%) **	45 (37.2)	18 (33.3)	27 (40.3)	0.431

Data are presented as number (%), median (interquartile range), or means ± standard deviation

* 187 patients included (76 normal MNA-SF score, 111 low MNA-SF score)

** 121 patients included (54 normal MNA-SF score, 67 low MNA-SF score)

BMI, body mass index; FFMI, fat-free mass index; TB, tuberculosis; COPD, chronic obstructive pulmonary disease; DM, diabetes mellitus; CV, cardiovascular disease; CT, computed tomography; AFB, acid-fast bacilli; CAT, chronic obstructive pulmonary disease assessment test; MNA-SF, Mini Nutritional Assessment Short Form; ASM, appendicular skeletal muscle mass; FMI, fat mass index; %EER, percentage estimated energy requirement; %RNI, percentage recommended nutrient intake; FVC, forced vital capacity

### Correlation between MNA-SF, body composition, disease severity, and nutrition intake

**Fig. 1:**
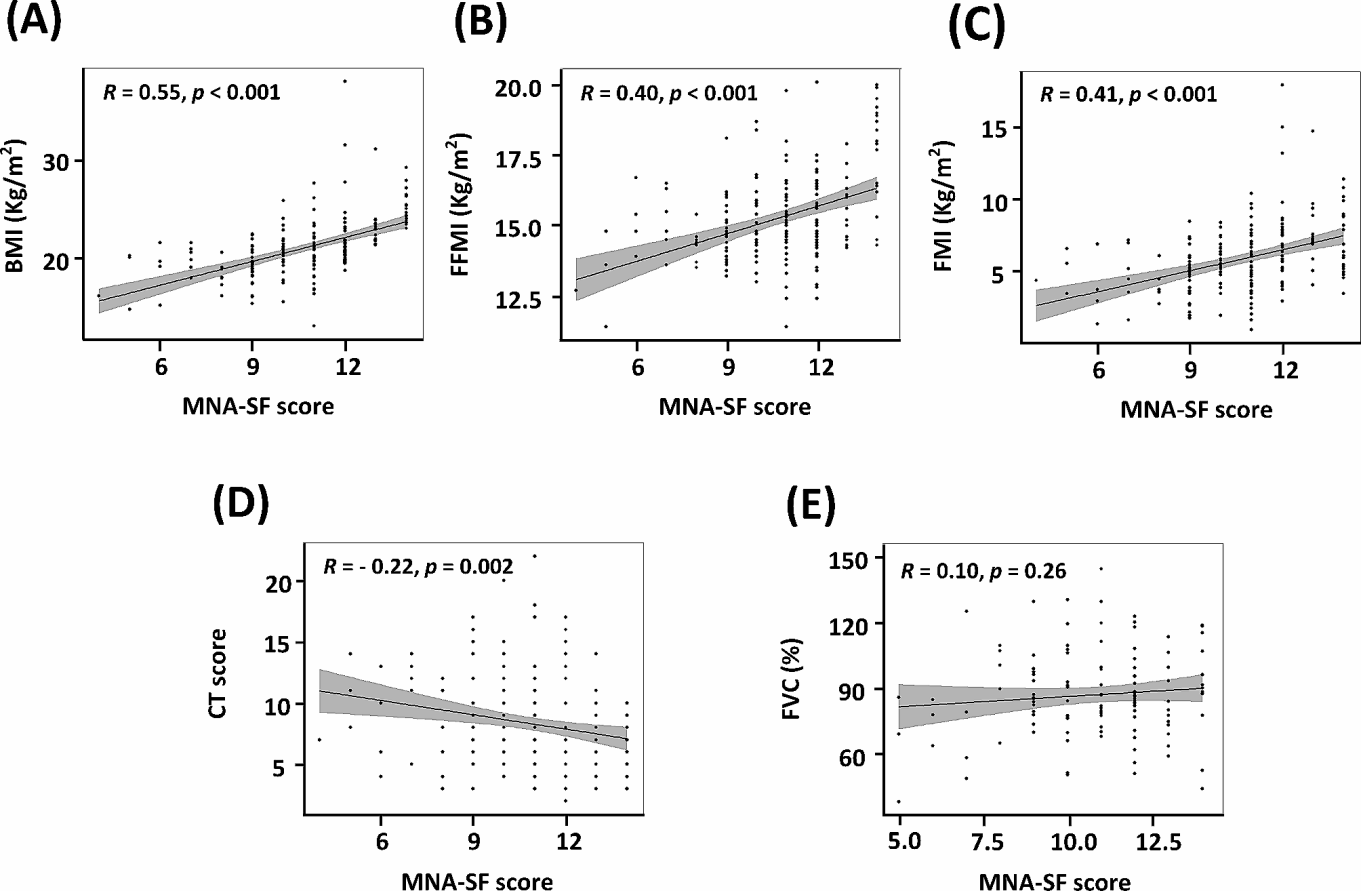
The correlations between MNA-SF and other nutritional measures, and between MNA-SF and disease severity. (**A**) Correlation between MNA-SF score and BMI. (**B**) Correlation plot between MNA-SF score and FFMI. (**C**) Correlation between MNA-SF score and FMI. (**D**) Correlation between MNA-SF and CT scores. (**E**) Correlation between MNA-SF score and FVC. MNA-SF, Mini Nutritional Assessment Short-Form; BMI, body mass index; FFMI, fat-free mass index; FMI, fat mass index; CT, computed tomography; FVC, forced vital capacity

The correlations between the MNA-SF score and other nutritional indicators are presented in Fig. 1. BMI (Fig. 1A), FFMI (Fig. 1B), and FMI (Fig. 1C) all demonstrated significant positive correlations with the MNA-SF score. In terms of disease severity, although the MNA-SF score was significantly negatively correlated with the CT score (Fig. 1D), there was no significant correlation between the MNA-SF score and forced vital capacity (FVC) (Fig. 1E). The correlations between the MNA-SF score and nutritional indicators and the MNA-SF score and disease severity analyzed according to sex are presented in Supplementary Figures S1 and S2. The correlation between the MNA-SF and CT scores was more prominent in females than in males (Supplementary Figure S2). No significant correlations were observed between the MNA-SF score and %EER or %RNI (Supplementary Figure S3).

### Relationship between NTM-PD radiographic severity and malnutrition

The correlation between MNA-SF scores and disease severity are presented in Table 3. Low MNA-SF scores showed a statistically significant association with CT scores ≥ 10 (aOR 2.72; 95% confidence interval [CI] 1.38–5.36). However, no significant associations were observed between low MNA-SF scores and high CAT scores, the presence of symptoms (cough, sputum, tightness), or FVC < 80%. Low MNA-SF scores were significantly associated with possible sarcopenia (aOR 2.37, 95% CI 1.12–5.00), sarcopenia (aOR 3.96, 95% CI 1.34–11.68), and adipopenia (aOR 11.04, 95% CI 3.72–32.75).

**Table 3:** Association between nutritional status and disease severity

		Normal MNA-SF		Low MNA-SF	
		95%CI	p-value	95%CI	p-value
Primary outcome	CT score ≥ 10	16 / 79 (20.3)		46 / 115 (40.0)	
	OR	1.0(ref)		2.63 (1.35–5.10)	0.004
	aOR†	1.0(ref)		2.72 (1.38–5.36)	0.004
Secondary outcome	Cough*	54 / 76 (71.1)		80 / 111 (72.1)	
	OR	1.0(ref)		1.05 (0.55–2.01)	0.879
	aOR†	1.0(ref)		1.00 (0.52–1.94)	0.992
	Sputum*	61 / 76 (80.3)		92 / 111 (82.9)	
	OR	1.0(ref)		1.19 (0.56–2.52)	0.648
	aOR†	1.0(ref)		1.21 (0.56–2.59)	0.628
	Tightness*	46 / 76 (60.5)		80 / 111 (72.1)	
	OR	1.0(ref)		1.68 (0.91–3.13)	0.099
	aOR†	1.0(ref)		1.70 (0.90–3.18)	0.100
	High CAT score*	40 / 76 (52.6)		71 / 111 (64.0)	
	OR	1.0(ref)		1.60 (0.88–2.89)	0.122
	aOR†	1.0(ref)		1.56 (0.85–2.85)	0.149
	FVC < 80%**	18 / 54 (33.3)		27 / 67 (40.3)	
	OR	1.0(ref)		1.35 (0.64–2.85)	0.431
	aOR†	1.0(ref)		1.45 (0.66–3.20)	0.359
	Possible sarcopenia	13 / 79 (16.5)		35 / 115 (30.4)	
	OR	1.0(ref)		2.22 (1.09–4.54)	0.029
	aOR†	1.0(ref)		2.37 (1.12–5.00)	0.024
	Sarcopenia	5 / 79 (6.3)		23 / 115 (20.0)	
	OR	1.0(ref)		3.70 (1.34–10.20)	0.011
	aOR†	1.0(ref)		3.96 (1.34–11.68)	0.013
	Adipopenia	4 / 79 (5.1)		41 / 115 (35.7)	
	OR	1.0(ref)		10.39 (3.54–30.46)	< 0.001
	aOR†	1.0(ref)		11.04 (3.72–32.75)	< 0.001

† Adjusted for age, sex, and smoking

* 187 patients included (76 normal MNA-SF score, 111 low MNA-SF score)

** 121 patients included (54 normal MNA-SF score, 67 low MNA-SF score)

MNA-SF, Mini Nutritional Assessment Short Form; CI, confidence interval; OR, odds ratio; aOR, adjusted odds ratio; CT, Computed tomography; CAT, chronic obstructive pulmonary disease assessment test; FVC, forced vital capacity

**Fig. 2:**
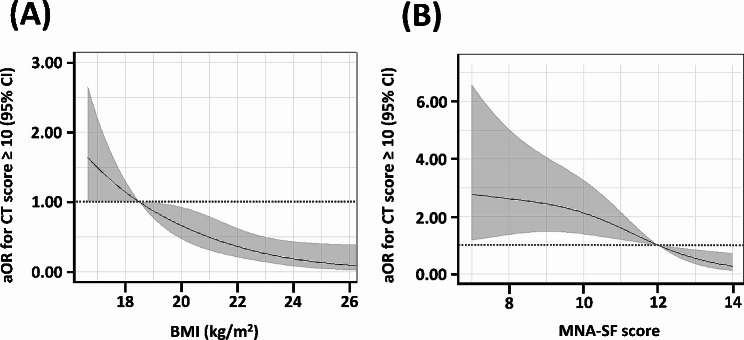
Relationship between radiological severity of nontuberculous mycobacterial pulmonary disease and nutrition. (**A**) Changes in the aOR for CT score ≥ 10 according to BMI. (**B**) Changes in the aOR for CT score ≥ 10 according to MNA-SF score. aOR, adjusted odds ratio; CI, confidence interval; CT, computed tomography; BMI, body mass index; MNA-SF, Mini Nutritional Assessment Short-Form

Figure 2 illustrates the changes in the aOR for disease severity with increasing BMI and MNA-SF scores, corresponding to nutritional indicators. As shown in Fig. 2A, the correlation with disease severity tended to decrease as BMI increased from the reference value of 18.5 kg/m^2^. For the MNA-SF score, a decrease below the normal threshold of 12 points was associated with an increase in the aOR for disease severity, whereas MNA-SF scores of > 12 points were associated with a lower aOR for disease severity (Fig. 2B).

## Discussion

This study investigated whether the MNA-SF could function as a nutritional screening tool in patients with NTM-PD. The correlations observed between the MNA-SF scores and established nutritional indicators, and the association between low MNA-SF scores and conditions linked to nutritional deficiencies, such as sarcopenia and adipopenia, confirmed its utility as a nutritional screening test. Furthermore, a decreased MNA-SF score was associated with higher CT scores, providing evidence of a correlation between nutritional status and disease severity.

The prevalence of nutritional inadequacy among patients with NTM-PD was 59.3% based on a threshold MNA-SF score of 12. Similarly, according to FFMI and applying the thresholds recommended by the European Society of Clinical Nutrition and Metabolism (male, FFMI < 17 kg/m^2^, female, FFMI < 15 kg/m^2^) [28] 62.4% of patients with NTM-PD exhibited nutritional deficiency in our study. Prior research using the same FFMI thresholds have reported nutritional deficiency rates of 27% for COPD and 28% for idiopathic pulmonary fibrosis [27, 29]. A previous study that used the same MNA-SF score threshold as the present study reported that 65% of patients with COPD were malnourished, whereas another study using MNA scores found that 19% of patients with idiopathic pulmonary fibrosis were at risk of malnutrition or malnourished [27, 30]. These findings indicate a relatively higher prevalence of nutritional deficiencies among patients with NTM-PD compared to that in patients with other respiratory diseases.

One of the main barriers frequently mentioned in conducting nutritional screening is the lack of time [31–33]. The MNA-SF used in this study is a shortened version of the MNA, a nutritional screening tool designed to assess the nutritional status of the elderly. It has been made more convenient for clinical use by reducing its length. While the complete MNA takes about 10 to 15 min to complete, the MNA-SF can be finished in under 5 min, making it an easier and more straightforward tool for clinical application [18, 34, 35]. Additionally, the MNA-SF has been known to be an effective clinical tool for identifying nutritional deficiencies across various conditions [36–38]. The previous study revealed that the MNA-SF is a fast, simple, and non-invasive tool with high patient compliance [34]. In our study, patients were provided with the MNA-SF questionnaire to complete independently. For those who had difficulty seeing due to presbyopia or understanding the questions, a research nurse assisted by reading and explaining the questions, ensuring all participants could complete the survey within 5 min. Unlike this study where healthcare professionals were available, in resource-limited settings general clinical staff may assist with the survey with additional nutritional education for the staff [34].

Previous studies have shown that a deterioration in nutritional status can be a risk factor for NTM-PD. A low BMI is associated with an increased incidence of NTM-PD [6] and low body fat and muscle mass are associated with NTM-PD development and mortality [39–41]. Our findings suggest that MNA-SF scores have significant moderate correlations with BMI, FFMI, FMI and associations with sarcopenia, possible sarcopenia, and adipopenia. These results suggest that MNA-SF scores effectively reflect the nutritional status of patients with NTM-PD. However, no significant correlation was observed between the MNA-SF score and dietary intake. Considering the lack of differences in total calorie and protein intakes observed between patients with mild and severe NTM-PD, as reported by Takayama et al. [8] dietary intake alone may not adequately reflect the nutritional status of patients with NTM-PD.

Our results showed that low MNA-SF scores were associated with CT scores ≥ 10. Similarly, Morimoto et al. reported a negative correlation between nutritional and CT scores in patients with NTM-PD [42]. However, in the present study, MNA-SF scores were not associated with CAT scores or FVC. Previous studies that investigated the usefulness of the MNA in patients with COPD found no correlation between CAT and MNA scores [43] however, lung function was correlated with MNA scores, in contrast to the present study [44]. Since the CAT was developed to assess the quality of life of patients with COPD, it may not adequately reflect the quality of life of patients with NTM-PD. In addition, pulmonary function is a more specific marker for COPD than for NTM-PD and may not therefore accurately reflect NTM-PD severity. For health-related quality of life (HR-QoL), CAT may have additional limitation in providing detailed insights into the specific characteristics of each HR-QoL domain. Although research on HR-QoL domains and nutrition in NTM-PD is limited, there are prior studies examining the correlation between nutritional status and HR-QoL domains in other chronic respiratory diseases such as COPD and bronchiectasis. For instance, Olveira et al. compared HR-QoL in bronchiectasis patients undergoing pulmonary rehabilitation, with one group receiving oral nutritional supplements enriched with beta-hydroxy-beta-methylbutyrate and the other group not receiving them. This study used the physical functioning domain from the Quality of Life-Bronchiectasis questionnaire (QOL-B). The findings showed significant improvement in HR-QoL only in the group taking the oral nutritional supplements, demonstrating a correlation between nutritional improvement and the physical function domain of HR-QoL [45]. In a study by Nguyen et al. involving COPD patients, the St George Respiratory Questionnaire, which includes symptom, activity, and impact scores, was used as the HR-QoL indicator. The results indicated that deteriorated nutritional status had the greatest impact on the activity score. While the impact score was also statistically significant, the symptom score did not show a significant correlation with nutritional status [46]. Therefore, it is considered necessary to investigate the relationship between nutritional status and specific domains of HR-QoL in patients with NTM-PD in the future.

The strength of this study is that it demonstrated the suitability of using the MNA-SF, a simple, convenient, and effective nutritional screening tool, in patients with NTM-PD. Furthermore, it revealed a correlation between the MNA-SF score and severity of NTM-PD as measured by CT. To appreciate our results correctly, we should consider the limitations of this study. First, this study assessed nutritional status at a single point in time as a cross-sectional study. To evaluate the impact of nutritional status more accurately on prognosis, a longitudinal assessment may be necessary. Therefore, future research should involve cohort studies with a temporal dimension or randomized controlled trial studies incorporating nutritional interventions. Second, the participants in this study were recruited from a single institution in East Asia; thus, the influence of regional differences cannot be entirely excluded. Previous studies have reported a deficiency in protein intake among the elderly population in East Asia [47, 48]. Considering this, while the median total protein Intake (% RNI) of 85.0% observed in our results might be characteristic of NTM-PD patients, we cannot rule out the influence of regional and cultural factors. Therefore, to overcome the limitations of this study, future research should consider recruiting participants from multiple institutions and countries. Third, there are limitations in the accuracy of body composition analysis through BIA. Unlike Dual-energy X-ray absorptiometry (DEXA), which measures body composition at an individual level, BIA estimates body composition using predictive equations, which can lead to prediction errors for individuals [49]. While studies exist for other chronic respiratory diseases such as COPD or bronchiectasis [50, 51], there is no research on this analysis for NTM-PD patients. Thus, the BIA used in our study may risk underestimating or overestimating fat-free mass and fat mass compared to the values measured by DEXA.

## Conclusions

Malnutrition is prevalent in patients with NTM-PD. The MNA-SF can effectively determine the nutritional status of patients with NTM-PD, and screening patients using the MNA-SF may contribute to the nutritional management of this population. Additionally, the correlation between the MNA-SF score and the radiographic severity of NTM-PD could serve as a starting point for research into whether nutritional status influences disease progression.

### Electronic supplementary material

Below is the link to the electronic supplementary material.

Supplementary Material 1

## Data Availability

The datasets used and/or analyzed during the current study are available from the corresponding author upon reasonable request.

## Abbreviations

aOR: Adjusted odds ratio
BMI: Body mass index
CAT: Chronic obstructive pulmonary disease assessment test
CI: Confidence interval
CT: Computed tomography
COPD: Chronic obstructive pulmonary disease
EER: Estimated energy requirement
FFMI: Fat-free mass index
FMI: Fat mass index
FVC: Forced vital capacity
BIA: Bioelectrical impedance analysis
IQR: Interquartile range
MNA: Mini Nutritional Assessment
MNA-SF: Mini Nutritional Assessment Short-Form
NTM-PD: Nontuberculous mycobacterial pulmonary disease
RNI: Reference nutrient intake
QOL-B: The Quality of Life-Bronchiectasis questionnaire
DEXA: Dual-energy X-ray absorptiometry
