# Supplementary material for: Usefulness of the mini nutritional assessment short-form for evaluating nutritional status in patients with nontuberculous mycobacterial pulmonary disease: a prospective cross-sectional study
Source: BMC Infect Dis. 2024 Jun 19;24:604. doi: 10.1186/s12879-024-09499-3 (PMC11186144; doi:10.1186/s12879-024-09499-3)
Supplement: Supplementary file 1 — Supplementary Material 1 [file 12879_2024_9499_MOESM1_ESM.docx]

**Supplementary Figure S1.** Relationship between MNA-SF score and other nutritional marker by sex.


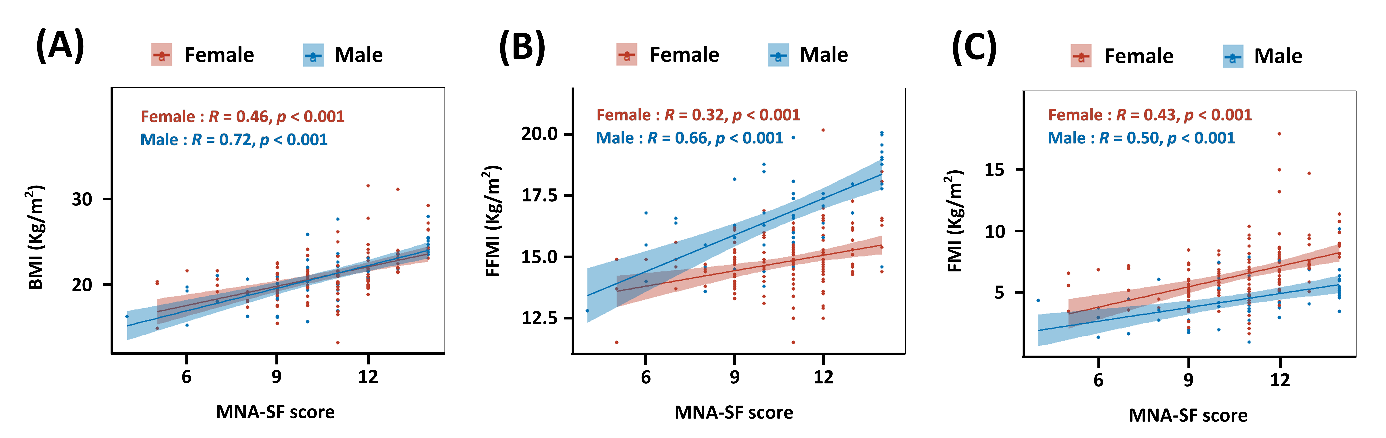


MNA-SF, mini nutritional assessment short-form; BMI, body mass index; FFMI, fat-free mass index; FMI, fat mass index.

**Supplementary Figure S2.** Relationship between MNA-SF score and disease severity by sex.


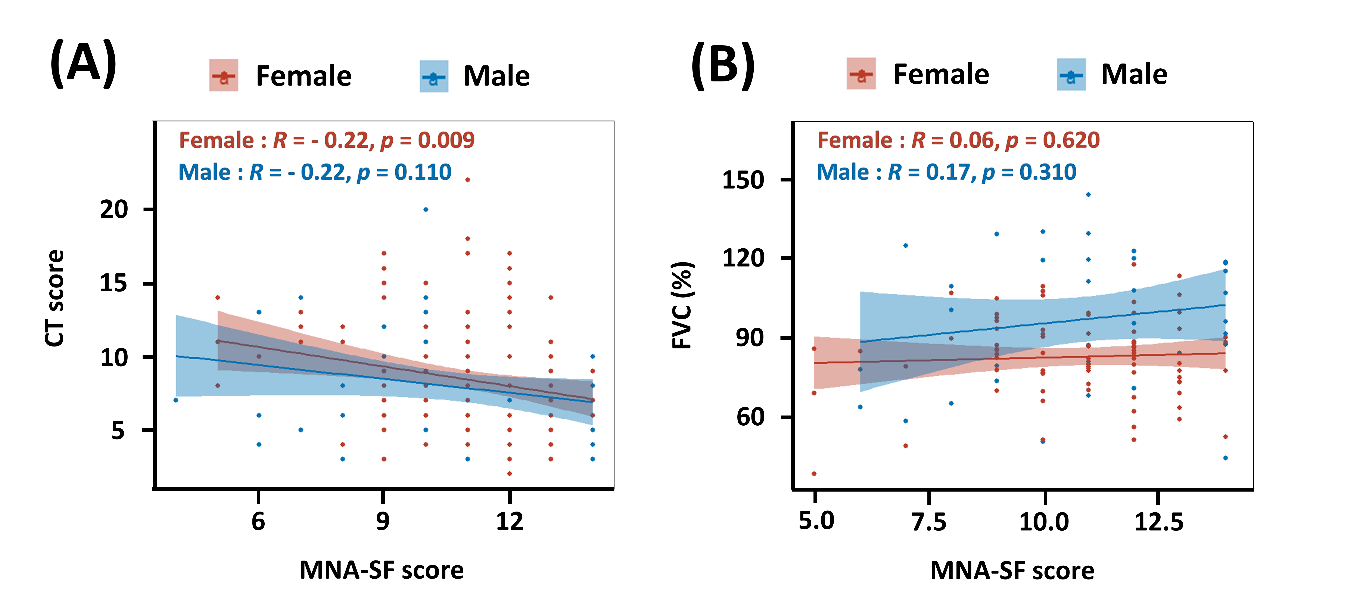


MNA-SF, mini nutritional assessment short-form; CT, computed tomography; FVC, forced vital capacity.

**Supplementary Figure S3.** Relationship between MNA-SF score and diet


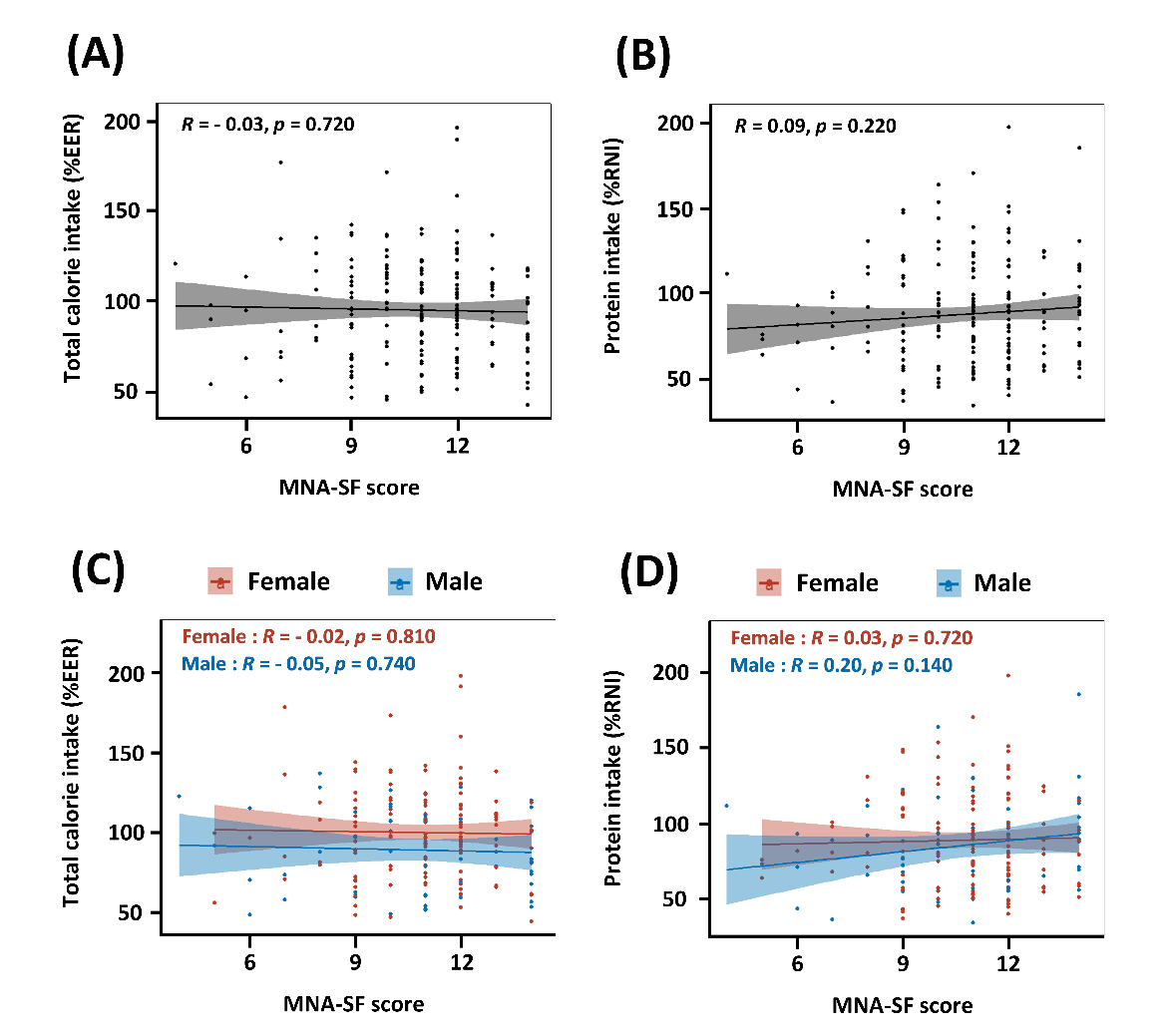


%EER, the percentiles of estimated energy requirement; %RNI, the percentiles of recommended nutrient intake; MNA-SF, mini nutritional assessment short-form.

**Supplementary Table S1.** MNA-SF scoring table

| **A. Has food intake declined over the past 3 months due to loss of appetite, digestive problems, chewing or swallowing difficulties?**  0 = severe decrease in food intake  1 = moderate decrease in food intake  2 = no decrease in food intake |
| --- |
| **B. Weight loss during the last 3 months**  0 = weight loss greater than 3 kg (6.6 lbs)  1 = does not know  2 = weight loss between 1 and 3 kg (2.2 and 6.6 lbs)  3 = no weight loss |
| **C. Mobility**  0 = bed or chair bound  1 = able to get out of bed / chair but does not go out  2 = goes out |
| **D. Has suffered psychological stress or acute disease in the past 3 months?**  0 = yes  2 = no |
| **E. Neuropsychological problems**  0 = severe dementia or depression  1 = mild dementia  2 = no psychological problems |
| **F1. Body Mass Index (BMI) (weight in kg) / (height in m)^2^**    0 = BMI less than 19  1 = BMI 19 to less than 21  2 = BMI 21 to less than 23  3 = BMI 23 or greater |
| If BMI is not available, replace question F1 with question F2.  Do not answer question F2 if question F1 is already completed. |
| **F2. Calf circumference (CC) in cm**  0 = CC less than 31  3 = CC 31 or greater |
| **Screening score (max. 14 points)**  12-14 points: Normal nutritional status  8-11 points: At risk of malnutrition  0-7 points: Malnourished |
